# Clinical outcomes of biodegradable polymer biolimus-eluting BioMatrix stents versus durable polymer everolimus-eluting Xience stents

**DOI:** 10.1371/journal.pone.0183079

**Published:** 2017-08-10

**Authors:** Da Hyon Lee, Taek Kyu Park, Young Bin Song, Woo Jung Chun, Rak Kyeong Choi, Jin-Ok Jeong, Eul Soon Im, Sang Wook Kim, Joo Myung Lee, Jeong Hoon Yang, Joo-Yong Hahn, Seung-Hyuk Choi, Jin-Ho Choi, Sang Hoon Lee, Hyeon-Cheol Gwon

**Affiliations:** 1 Division of Cardiology, Department of Internal Medicine, Heart Vascular Stroke Institute, Samsung Medical Center, Sungkyunkwan University School of Medicine, Seoul, Republic of Korea; 2 Division of Cardiology, Department of Internal Medicine, Samsung Changwon Hospital, Sungkyunkwan University School of Medicine, Changwon, Republic of Korea; 3 Sejong General Hospital, Bucheon, Republic of Korea; 4 Chungnam National University Hospital, Daejeon, Republic of Korea; 5 Dongsuwon General Hospital, Suwon, Republic of Korea; 6 Chung-Ang University Hospital, Seoul, Republic of Korea; University of Tampere, FINLAND

## Abstract

There are limited data about clinical outcomes of biodegradable polymer biolimus-eluting BioMatrix stents (BP-BES) and durable polymer everolimus-eluting Xience stents (DP-EES) in real world practice. We sought to compare the clinical outcomes of BP-BES and DP-EES in real world cohorts of patients undergoing percutaneous coronary intervention. A prospective multicenter registry enrolled 999 patients treated with BP-BES and 1,000 patients treated with DP-EES. The primary outcome was target lesion failure, defined as a composite of cardiac death, target vessel-related myocardial infarction, or target lesion revascularization. Definite or probable stent thrombosis was also compared in total and propensity score-matched cohorts. The median follow-up duration was 24 months, and mean age was 65 years (interquartile range, 56–72 years). Patients receiving BP-BES had a lower prevalence of acute coronary syndrome, prior myocardial infarction, multi-vessel disease, bifurcation lesions, and left anterior descending artery lesions than those receiving DP-EES. After propensity score matching (692 pairs), target lesion failure occurred in 22 patients receiving BP-BES and in 25 patients receiving DP-EES (3.2% versus 3.6%; adjusted hazard ratio [HR], 0.92; 95% confidence interval [CI], 0.53 to 1.60; p = 0.77). The risk of definite or probable stent thrombosis did not differ between the 2 groups (0.4% versus 0.4%; adjusted HR, 1.03; 95% CI, 0.21 to 4.98; p = 0.97). The results were consistent across various subgroups. In the propensity score-matched analysis of real world cohorts, BP-BES showed similar clinical outcomes compared to DP-EES. We need to investigate about whether differences in clinical outcome emerge during long-term follow-up.

## Introduction

Drug-eluting stents (DES) consisting of a metal platform and a polymer coating with the release of antiproliferative drugs had reduced restenosis compared with bare metal stents [1,2]. However, first-generation DES such as sirolimus- or paclitaxel-eluting stent also increased the concern about late stent thrombosis [3–5]. Durable polymer was potentially associated with delayed healing, allergic reaction, and chronic inflammation that could lead to impaired endothelialization of the stent strut and positive vessel remodeling, and increase the risk of stent thrombosis [6,7]. Newer generation durable polymer-coated DES was developed to improve polymer biocompatibility, and biocompatible durable polymer everolimus-eluting stent (DP-EES) is regarded due to safety and efficacy profile as the gold standard [8]. In addition, newer generation DES using biodegradable polymer was also developed to overcome the long-term adverse vascular reaction related to the durable polymer. Recent studies showed that biodegradable polymer biolimus-eluting stent (BP-BES) had a safety benefit compared with first-generation DES in terms of a reduction in very late stent thrombosis [9]. In addition, BP-BES showed similar safety and efficacy in comparison to DP-EES [10,11]. Although previous studies were randomized controlled trials which were optimized to compare the safety and efficacy between two groups, they might be inappropriate to show directly applicable data to real world, because of several inclusion and exclusion criteria. Therefore, we sought to compare the safety and efficacy of BP-BES and DP-EES in an “all-comer” registry.

## Materials and methods

### Study population

**Fig 1:**
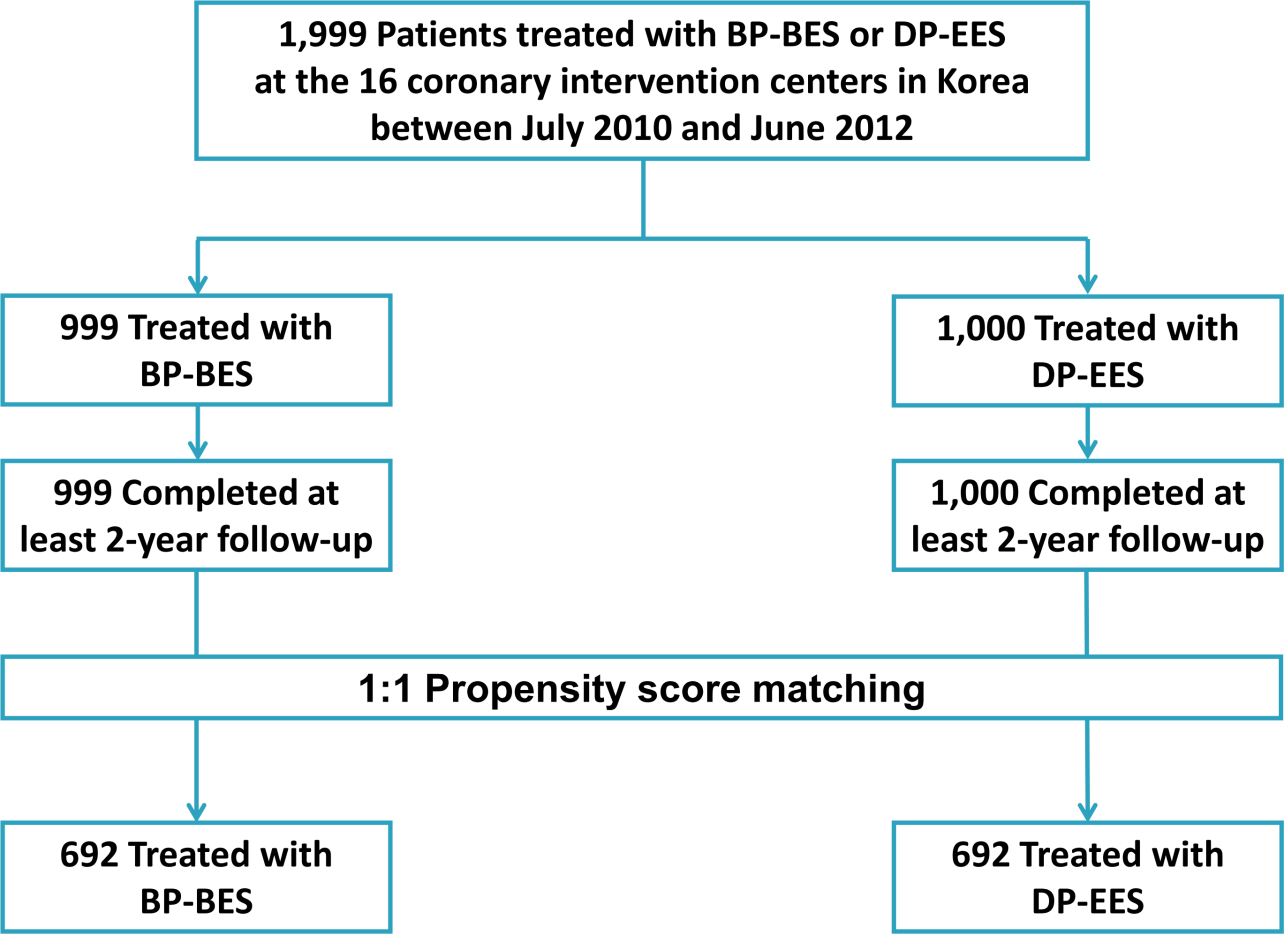
Study population. BP-BES = biodegradable polymer biolimus-eluting stent; DP-EES = durable polymer everolimus-eluting stent.

A total of 1,999 patients who underwent percutaneous coronary intervention (PCI) with BP-BES (BioMatrix Flex, Biosensors Inc, Newport Beach, CA, USA) or DP-EES (Xience V or Prime, Abbott Vascular, Santa Clara, CA, USA) at the 16 coronary intervention centers in Korea between July 2010 and June 2012 were enrolled in the prospective multicenter registry (Fig 1). All patients older than 20 years who underwent PCI with DES were registered without any restrictions for number, location, size and length of treated lesions. Patients with cardiogenic shock were excluded. This study was approved by the Institutional Review Board of Samsung Medical Center, Samsung Changwon Hospital, Sejong General Hospital, Chungnam National University Hospital, Dongsuwon General Hospital, Chung-Ang University Hospital, Sam General Hospital, Kyungpook National University Hospital, Kangbuk Samsung Hospital, Kangwon National University Hospital, Seoul Medical Center, Inje University Ilsan Paik Hospital, S-Jung-Ang Hospital, KEPCO Medical Center, Konkuk University Medical Center, and Kyung Hee University Hospital. All patients provided written informed consent for access to an institutional registry.

### Procedure and medical treatment

All interventions were performed according to current practice guideline. All patients received loading doses of aspirin (300 mg) and clopidogrel (300–600 mg) before PCI unless antiplatelet medications had previously been prescribed. Unfractionated heparin was administered during PCI in order to achieve an activated clotting time of 250–300 seconds. The choice of DES type and duration of dual antiplatelet therapy was at the operator’s discretion.

### Study outcomes and definitions

The primary outcome was target lesion failure (TLF) after stent implantation, defined as a composite of cardiac death, target vessel-related myocardial infarction, or target lesion revascularization. Individual components of the primary outcome, all-cause death, myocardial infarction, and definite or probable stent thrombosis were compared as the secondary outcome. All deaths were considered cardiac unless a definite non-cardiac cause could be established. Myocardial infarction was defined as elevated cardiac enzymes (troponin or myocardial band fraction of creatine kinase) greater than the upper limit of the normal value that occurred alongside ischemic symptoms or electrocardiography findings indicative of ischemia unrelated to the index procedure. Target lesion revascularization was defined as revascularization within stent or within 5mm border of stent deployment. Definite or probable stent thrombosis was assessed according to the definition of the Academic Research Consortium (9). Basal clinical and angiographic characteristics and all follow-up data were collected prospectively into a dedicated PCI registry, and missed additional clinical information was obtained by the review of medical record or telephone encounter.

### Statistical analyses

Continuous variables were expressed as mean ± SD and compared using an independent t-test or the Mann-Whitney test. Categorical variables were summarized as numbers with percentages and compared using chi-square test or Fisher’s exact test. Time-to-event hazard curves were presented with Kaplan-Meier estimates and were compared using a log-rank test. To balance the patients for various clinical and angiographic characteristics, we used the propensity score matching method in a pairwise manner. The propensity score, which represents the probability of the use of BP-BES, was estimated without regard to outcome using multiple logistic regression analysis [12]. The pairs were matched using one-to-one individual matching between the BP-BES and DP-EES group. The balance was deemed satisfactory when the standardized mean differences are less than 10%. In the propensity score-matched population, the reduction in the risk of an outcome was compared using a clustered Cox regression model [13]. All tests were two-tailed, and p values less than 0.05 were considered significant. R software version 3.3.2 (R Foundation for Statistical Computing, Vienna, Austria) was used for statistical analysis.

## Results

### Baseline characteristics

Among a total of 1,999 patients (2,687 lesions), 999 patients (1,258 lesions) underwent PCI using BP-BES and 1,000 patients (1,429 lesions) underwent PCI using DP-EES. Mean age was 65 years (interquartile range, 56–72 years). Baseline clinical and angiographic characteristics of patients were described in Table 1, and angiographic characteristics of lesions were described in Table 2. Patients treated with DP-EES had a higher prevalence of acute coronary syndrome at admission, dyslipidemia, previous myocardial infarction, and previous bypass surgery than those treated with BP-BES. Multi-vessel disease, bifurcation lesion, and ACC/AHA lesion type B2 or C were more common in the DP-EES group than in the BP-BES group. The stent was implanted more and longer in DP-EES group than BP-BES group. After propensity score matching, 692 pairs were yielded, and baseline characteristics were well-balanced between the 2 groups (Table 1).

**Table 1:** Baseline characteristics.

	Total population				Propensity score-matched population			
	BP-BES (n = 999)	DP-EES (n = 1,000)	p Value	SMD (%)	BP-BES (n = 692)	DP-EES (n = 692)	p Value	SMD (%)
Age, years	64.2 ± 10.8	63.7 ± 11.2	0.28	-4.6	64.1 ± 10.6	63.9 ± 11.2	0.69	-1.8
Male	695 (69.6)	687 (68.7)	0.71	-1.9	487 (70.4)	484 (69.9)	0.91	-0.9
Clinical presentation			0.002				0.90	
SIHD	473 (47.3)	397 (39.7)		-15.5	308 (44.5)	301 (43.5)		-2.0
NSTE-ACS	378 (37.8)	447 (44.7)		13.9	282 (40.8)	290 (41.9)		2.3
STEMI	148 (14.8)	156 (15.6)		2.2	102 (14.7)	101 (14.6)		-0.4
Coexisting conditions								
Diabetes mellitus	306 (30.6)	330 (33.0)	0.28	5.1	216 (31.2)	216 (31.2)	1.00	0.0
Hypertension	604 (60.5)	559 (55.9)	0.04	-9.3	411 (59.4)	395 (57.1)	0.40	-4.7
Dyslipidemia	377 (37.7)	466 (46.6)	<0.001	17.8	290 (41.9)	303 (43.8)	0.51	3.8
Chronic Renal failure	15 (1.5)	15 (1.5)	1.00	0.0	10 (1.4)	8 (1.2)	0.81	-2.4
Risk factors								
Current smoker	283 (28.3)	260 (26.0)	0.26	-5.2	190 (27.5)	183 (26.5)	0.71	-2.3
Previous MI	42 (4.2)	74 (7.4)	0.003	12.2	37 (5.3)	37 (5.3)	1.00	0.0
Previous PCI	107 (10.7)	134 (13.4)	0.08	7.9	91 (13.2)	87 (12.6)	0.80	-1.7
Previous CABG	13 (1.3)	27 (2.7)	0.04	8.6	12 (1.7)	11 (1.6)	1.00	-0.9
Previous stroke	76 (7.6)	38 (3.8)	<0.001	-19.9	35 (5.1)	35 (5.1)	1.00	0.0
LVEF, %*	57.7 ± 12.6	58.1 ± 12.0	0.80	3.5	58.1 ± 12.3	58.3 ± 11.8	0.78	2.2
Angiographic disease extent			<0.001				0.85	
1 vessel disease	534 (53.5)	414 (41.4)		-24.6	324 (46.8)	327 (47.3)		1.0
2 vessel disease	306 (30.6)	338 (33.8)		6.8	236 (34.1)	227 (32.8)		-2.8
3 vessel disease	159 (15.9)	248 (24.8)		20.6	132 (19.1)	138 (19.9)		2.0
No. of treated lesion per patients	1.4 ± 0.6	1.5 ± 0.8	<0.001	21.9	1.4 ± 0.7	1.4 ± 0.6	0.90	-2.6
Treated vessel								
Left main	21 (2.1)	29 (2.9)	0.32	4.8	18 (2.6)	21 (3.0)	0.75	2.3
Left anterior descending	540 (54.1)	625 (62.5)	<0.001	17.4	397 (57.4)	410 (59.2)	0.50	3.7
Left circumflex	287 (28.7)	233 (23.3)	0.007	-12.8	187 (27.0)	156 (22.5)	0.06	-10.8
Right coronary	334 (33.4)	364 (36.4)	0.18	6.2	240 (34.7)	237 (34.2)	0.91	-1.1
Any AHA/ACC B2 or C	583 (61.2)	697 (75.1)	<0.001	32.1	467 (67.5)	476 (68.8)	0.63	2.8
Any bifurcation	198 (20.5)	257 (25.7)	0.007	11.9	163 (23.6)	167 (24.1)	0.85	1.2
Any thrombotic lesion	135 (14.0)	156 (15.6)	0.35	4.4	102 (14.7)	111 (16.0)	0.56	3.5
Any calcified lesion	192 (19.9)	187 (18.7)	0.53	-3.1	136 (19.7)	136 (19.7)	1.00	0.0
No. of stents per patient	1.3 ± 0.7	1.5 ± 0.8	<0.001	20.1	1.4 ± 0.7	1.4 ± 0.6	0.92	-4.7
Total stent length, mm	29.2 ± 16.3	37.5 ± 21.9	<0.001	38.3	31.3 ± 17.4	31.6 ± 15.8	0.71	0.9
Maximal stent diameter, mm	3.2 ± 0.5	3.2 ± 0.4	0.33	-6.4	3.2 ± 0.5	3.2 ± 0.4	0.68	0.8
Multivessel PCI	166 (16.6)	221 (22.1)	0.002	13.3	135 (19.5)	120 (17.3)	0.29	-5.8

Values are expressed as mean ± SD or n (%). ACC/AHA = American College of Cardiology/American Heart Association; BP-BES = biodegradable polymer biolimus-eluting stent; CABG = coronary artery bypass graft; DP-EES = durable polymer everolimus-eluting stent; LVEF = left ventricular ejection fraction; NSTE-ACS = non-ST-segment elevation acute coronary syndrome; MI = myocardial infarction; PCI = percutaneous coronary intervention; SIHD = stable ischemic heart disease; SMD = standardized mean difference; STEMI = ST-segment elevation myocardial infarction.

*LVEF was available in 767 patients (76.8%) with BP-BES and 831 patients (83.1%) with DP-EES in total population, 539 patients (77.9%) with BP-BES and 574 patients (82.9%) with DP-EES in propensity score-matched population.

**Table 2:** Angiographic characteristics of lesions in total population.

	BP-BES (n = 1,258)	DP-EES (n = 1,429)	p Value
Target vessel location			0.003
Left main	21/1,258 (1.7)	29/1,429 (2.0)	
Left anterior descending	570/1,258 (45.3)	707/1,429 (49.5)	
Left circumflex	296/1,258 (23.5)	255/1,429 (17.8)	
Right coronary	371/1,258 (29.5)	438/1,429 (30.7)	
ACC/AHA lesion class			<0.001
A	132/1,202 (11.0)	79/1,309 (6.0)	
B1	375/1,202 (31.2)	331/1,309 (25.3)	
B2	292/1,202 (24.3)	239/1,309 (18.3)	
C	403/1,202 (33.5)	660/1,309 (50.4)	
Type B2 or C lesions	695/1,202 (57.8)	899/1,309 (68.7)	<0.001
Bifurcation	213/1,219 (17.5)	290/1,428 (20.3)	0.07
Thrombus present	144/1,218 (11.8)	164/1,427 (11.5)	0.84
Calcification	229/1,218 (18.8)	230/1,428 (16.1)	0.08
Maximum pressure deployment, atm	12.7 ± 4.0 (1,164)	13.3 ± 3.9 (1,392)	0.007
Maximal stent diameter per lesion, mm	3.1 ± 0.5 (1,258)	3.0 ± 0.4 (1,429)	0.005
Total stent length per lesion, mm	23.3 ± 7.8 (1,258)	26.8 ± 10.0 (1,429)	<0.001
No. of stent per lesion	1.1 ± 0.2 (1,258)	1.1 ± 0.2 (1,429)	0.56

Values are expressed as mean ± SD (number of lesions assessed) or number of lesions/number of lesions assessed (%). ACC/AHA = American College of Cardiology/American Heart Association; BP-BES = biodegradable polymer biolimus-eluting stent; DP-EES = durable polymer everolimus-eluting stent; PCI = percutaneous coronary intervention.

### Clinical outcomes of the total population

The median follow-up was 24 months (interquartile range, 24–32 months) in the BP-BES group and 24 months (interquartile range, 24–33 months) in the DP-EES group. The cumulative outcomes are summarized in Table 3. The TLF occurred in 25 patients treated with BP-BES and in 39 patients treated with DP-EES (2.5% versus 3.9%, p = 0.12). There were no significant differences between 2 groups with respect to all-cause death, cardiac death, myocardial infarction, target vessel-related myocardial infarction, target lesion revascularization.

**Table 3:** Clinical outcomes in the total and propensity score-matched populations.

	BP-BES (n = 999)	DP-EES (n = 1,000)	Hazard Ratio (95% CI)	p Value
Total population				
Target lesion failure	25 (2.5)	39 (3.9)	0.67 (0.41–1.11)	0.12
All-cause death	45 (4.5)	38 (3.8)	1.23 (0.80–1.89)	0.35
Cardiac death	17 (1.7)	22 (2.2)	0.81 (0.43–1.52)	0.51
Myocardial infarction	9 (0.9)	13 (1.3)	0.72 (0.31–1.70)	0.46
Target vessel-related myocardial infarction	2 (0.2)	6 (0.6)	0.35 (0.07–1.73)	0.20
Target lesion revascularization	8 (0.8)	17 (1.7)	0.50 (0.22–1.17)	0.11
Definite or probable stent thrombosis	4 (0.4)	4 (0.4)	1.01 (0.25–4.05)	0.99
Propensity score-matched population	(n = 692)	(n = 692)		
Target lesion failure	22 (3.2)	25 (3.6)	0.92 (0.53–1.60)	0.77
All-cause death	29 (4.2)	25 (3.6)	1.20 (0.70–2.05)	0.50
Cardiac death	15 (2.2)	14 (2.0)	1.11 (0.53–2.31)	0.78
Myocardial infarction	9 (1.3)	12 (1.7)	0.80 (0.34–1.89)	0.61
Target vessel-related myocardial infarction	2 (0.3)	5 (0.7)	0.43 (0.09–2.16)	0.31
Target lesion revascularization	7 (1.0)	11 (1.6)	0.70 (0.27–1.80)	0.46
Definite or probable stent thrombosis	3 (0.4)	3 (0.4)	1.03 (0.21–4.98)	0.97

Values are expressed as n (%). BP-BES = biodegradable polymer biolimus-eluting stent; CI = Confidence interval; DP-EES = durable polymer everolimus-eluting stent; HR = hazard ratio; MI = myocardial infarction.

**Fig 2:**
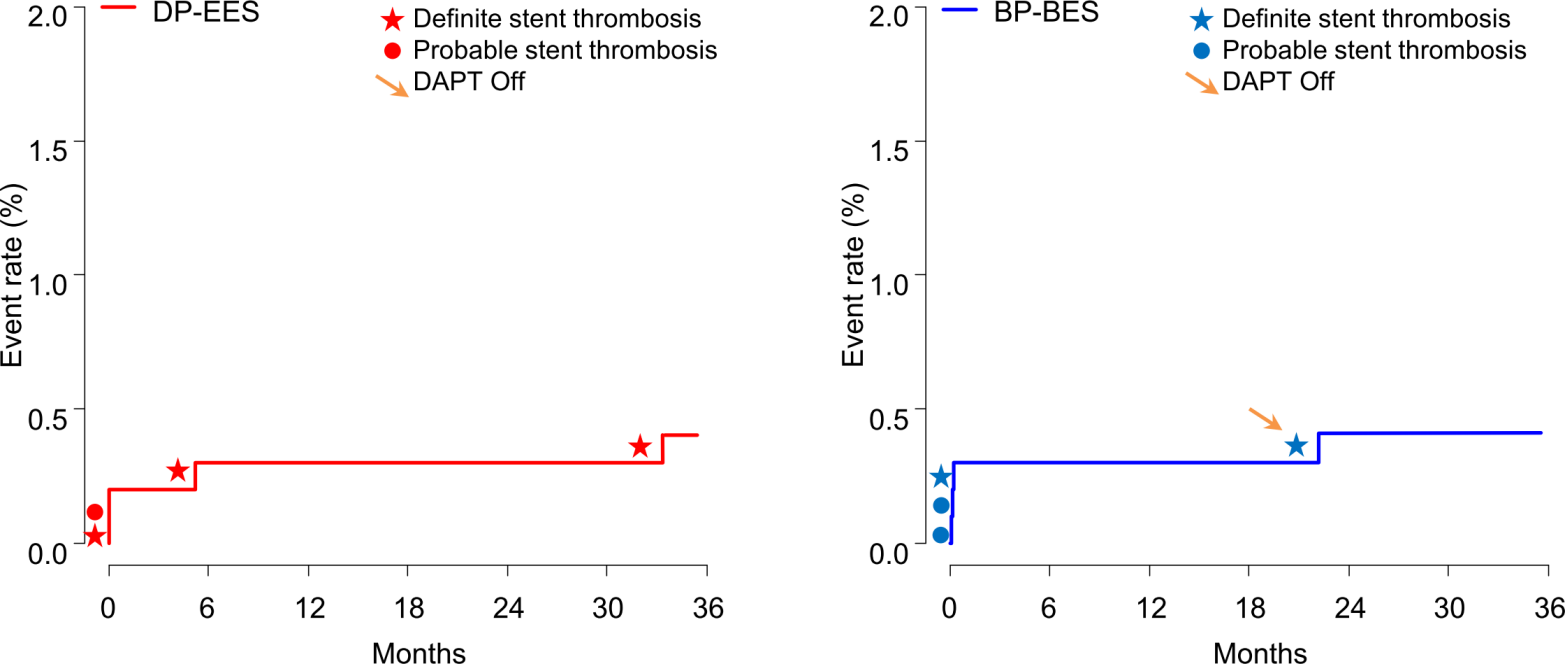
Kaplan-Meier curves for definite or probable stent thrombosis. BP-BES = biodegradable polymer biolimus-eluting stent; DAPT = dual antiplatelet therapy; DP-EES = durable polymer everolimus-eluting stent.

The definite or probable stent thrombosis occurred in 4 patients treated with BP-BES and in 4 patients treated with DP-EES (0.4% versus 0.4%, p = 0.99, Table 4, Fig 2). All except 2 patients were on dual antiplatelet therapy at the time of the event. Two patients presented with very late stent thrombosis at 675 days and 1,015 days after index procedure, respectively (Table 5). The administration of dual antiplatelet therapy was continued similarly between the 2 groups at 1 year (83.4% versus 85.5%, p = 0.23), but the use of dual antiplatelet therapy maintained more frequently in BP-BES group than in DP-EES group at 2 years (44.0% versus 33.6%, p <0.001).

**Table 4:** Stent thrombosis and use of dual antiplatelet therapy in the total and propensity score-matched populations.

	Total population			Propensity score-matched population		
	BP-BES (n = 999)	DP-EES (n = 1,000)	p Value	BP-BES (n = 692)	DP-EES (n = 692)	p Value
Definite						
Acute (<1 day)	-	1 (0.1)	-	-	1 (0.1)	-
Subacute (1–30 days)	1 (0.1)	1 (0.1)	>0.99	1 (0.1)	1 (0.1)	>0.99
Late (31–365 days)	-	-	-	-	-	-
Very late (≥366 days)	1 (0.1)	1 (0.1)	-	1 (0.1)	1 (0.1)	-
Probable						
Acute (<1 day)	-	1 (0.1)	-	-	-	-
Subacute (1–30 days)	2 (0.2)	-	-	1 (0.1)	-	-
Late (31–365 days)	-	-	-	-	-	-
Very late (≥366 days)	-	-	-	-	-	-
Definite or probable stent thrombosis	4 (0.4)	4 (0.4)	>0.99	3 (0.4)	3 (0.4)	>0.99

Values are expressed as n (%). BP-BES = biodegradable polymer biolimus-eluting stent; DP-EES = durable polymer everolimus-eluting stent.

**Table 5:** Detailed description of definite or probable stent thrombosis.

	**Gender/Age**	**Initial presentation**	**Target vessel**	**Days after procedure to event**	**Events**	**Days after discontinuation of DAPT to event**	**Antiplatelet agent at event**
BP-BES group							
Definite	F/79	Stable angina	LCx, RCA	7	MI, TLR	-	A+C
Definite	M/72	Stable angina	RCA	675	Cardiac death, MI	598	A
Probable	M/75	STEMI	RCA	2	Cardiac death	-	A+C
Probable	F/79	Stable angina	LAD	5	Cardiac death	-	A+C
DP-EES group							
Definite	F/83	STEMI	LM	1	Cardiac death, MI, TLR	-	A+C
Definite	F/69	Stable angina	LAD	158	Cardiac death, MI, TLR	-	A+C
Definite	M/70	STEMI	RCA	1015	Cardiac death, MI	639	A
Probable	M/88	NSTEMI	RCA	1	Cardiac death, MI	-	A+C

A = aspirin; BP-BES = biodegradable polymer biolimus-eluting stent; C = clopidogrel; F = female; DAPT = dual antiplatelet therapy; DP-EES = durable polymer everolimus-eluting stent; M = male; MI = myocardial infarction; NSTEMI = non-ST-segment elevation myocardial infarction; LAD = left anterior descending artery; RCA = right coronary artery; STEMI = ST-segment elevation myocardial infarction; TLR = target lesion revascularization.

### Clinical outcomes of the propensity score-matched population

**Fig 3:**
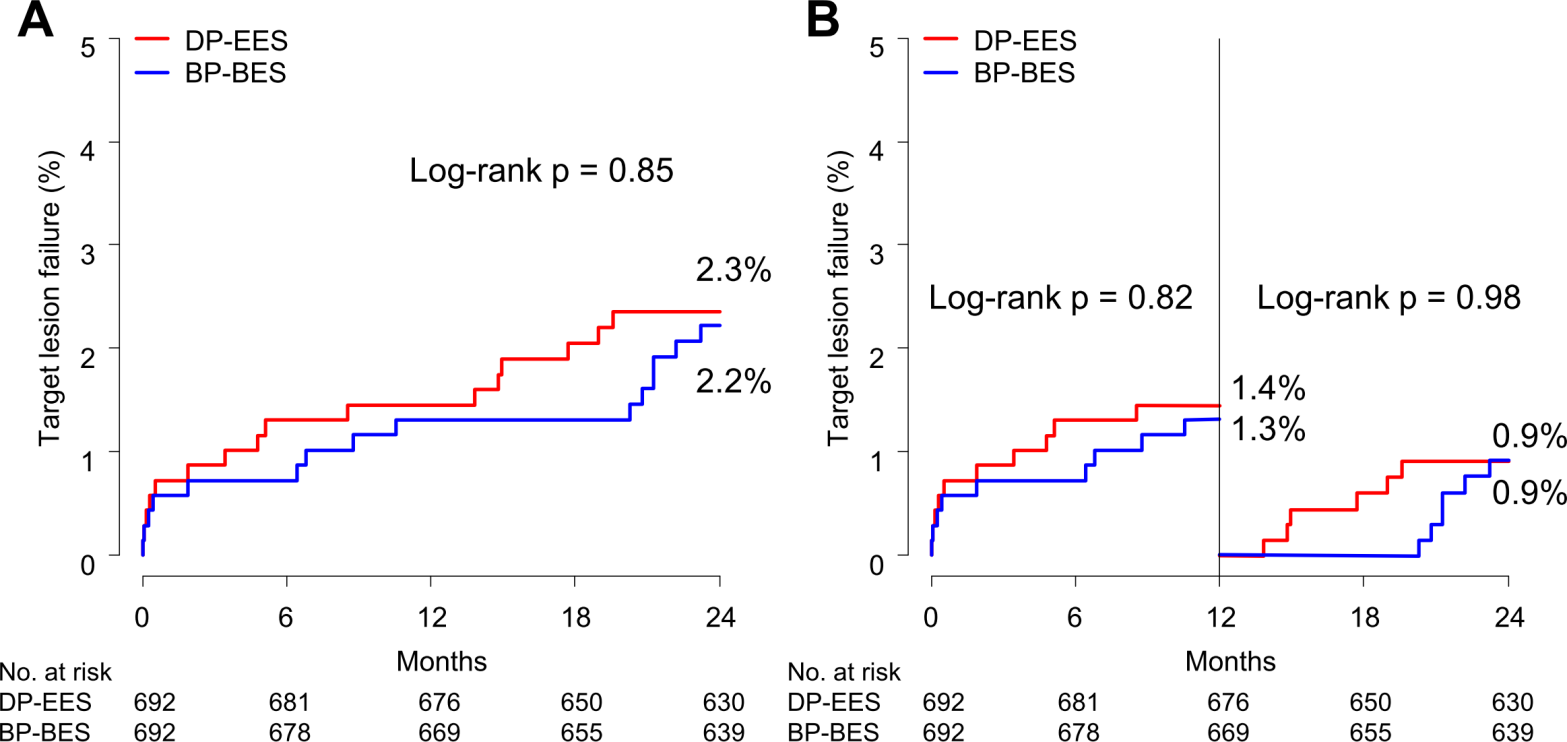
Kaplan-Meier curves for clinical outcomes in the propensity score-matched cohort. (A) Target lesion failure. (B) Target lesion failure at 1-year landmark. There were no significant differences of clinical outcomes between 2 groups. BP-BES = biodegradable polymer biolimus-eluting stent; DP-EES = durable polymer everolimus-eluting stent.

Fig 3 shows Kaplan-Meier curves for the TLF in the propensity score-matched population. The cumulative rate of TLF was similar in the 2 groups (3.2% versus 3.6%, p = 0.77, Table 3) and no significant differences were observed in the rate of TLF at 1 year and in a landmark analysis between 1 year and 2 years (Fig 3B). Definite or probable stent thrombosis was very low and comparable between both groups (0.4% versus 0.4%, p = 0.97).

### Subgroup analysis of the total population

**Fig 4:**
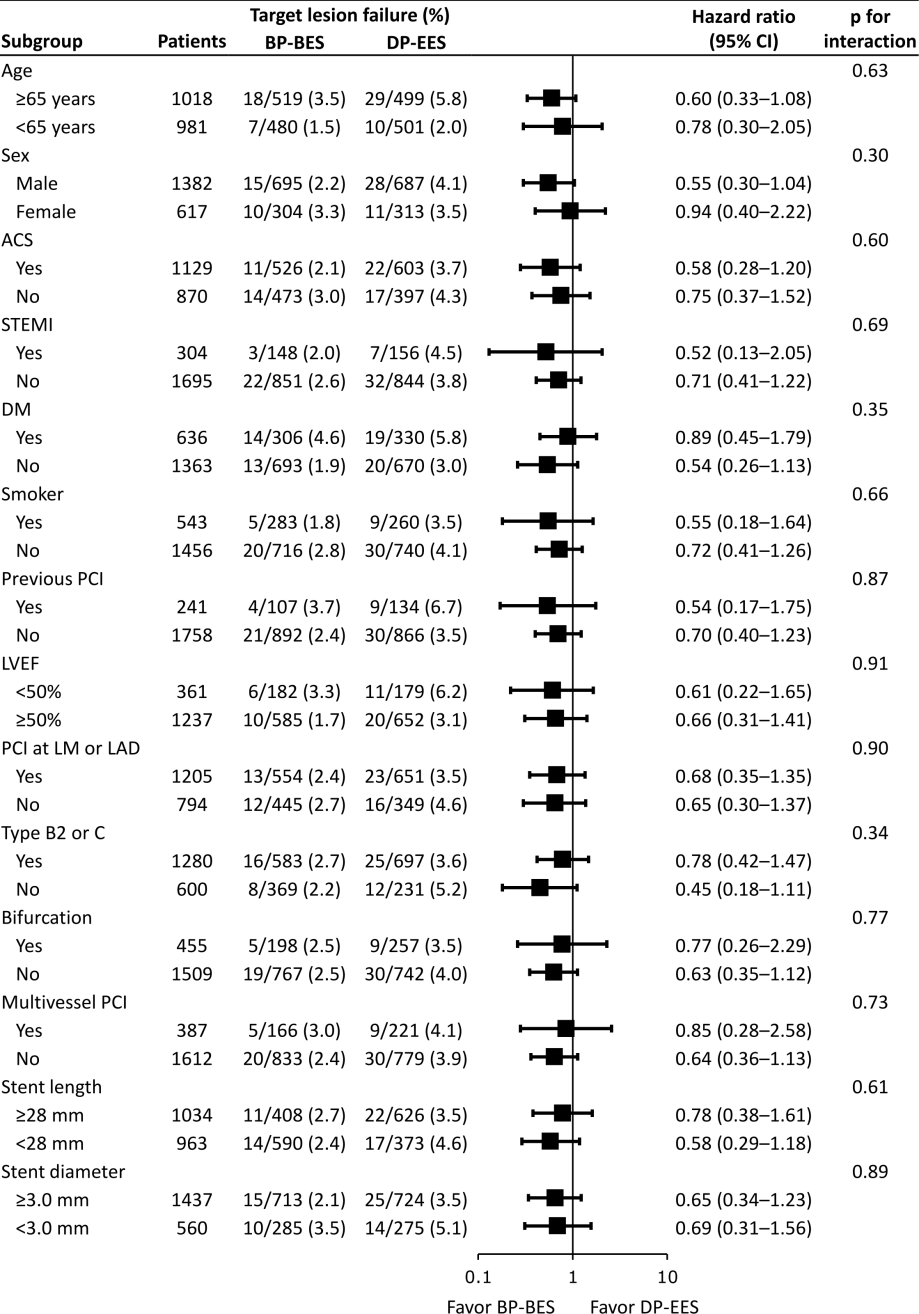
Hazard ratios for target lesion failure according to various subgroups in the total population. Hazard ratios for target lesion failure in BP-BES were compared with DP-EES in various subgroups. There were no significant interactions between target lesion failure and subgroups. ACC/AHA = American College of Cardiology/ American Heart Association; ACS = acute coronary syndrome; BP-BES = biodegradable polymer biolimus-eluting stent; DM = diabetes mellitus; DP-EES = durable polymer everolimus-eluting stent; LAD = left anterior descending coronary artery; LM = left main coronary artery; LVEF = left ventricular ejection fraction; PCI = percutaneous coronary intervention; STEMI = ST-elevation myocardial infarction.

Hazard ratios for the TLF according to several clinical, angiographic or procedural subgroups were shown in Fig 4. There were no significant interactions between TLF and subgroups.

## Discussion

The prospective observational registry provides head-to-head comparison of BP-BES versus DP-EES, and includes all-comers except for cardiogenic shock. Present data show that, BP-BES had similar safety and efficacy profiles in the total and propensity score-matched populations. TLF rate did not differ significantly between the 2 groups, and individual outcome also is not different. The rate of probable or definite stent thrombosis was very low and similar between the 2 groups.

BP-BES was demonstrated to have an equivalent or superior efficacy and safety in the final 5-year report of the LEADERS (Limus Eluted From A Durable Versus ERodable Stent Coating) trial compared with durable polymer sirolimus-eluting stents [9]. Especially, very late stent thrombosis was significantly lower in patients treated with BP-BES than in those treated with durable polymer-coated sirolimus-eluting stents. However, these advantages of BP-BES have not been observed in comparison with DP-EES. Two randomized trials, COMPARE II (Comparison of the Everolimus Eluting With the Biolimus A9 Eluting Stent) and NEXT (NOBORI Biolimus-Eluting Versus XIENCE/PROMUS Everolimus-Eluting Stent Trial) reported that BP-BES was not superior in terms of safety and efficacy compare with DP-EES at 1 year [14,15]. On the contrary, network meta-analyses have shown an increased risk of BP-BES regarding to MI or stent thrombosis, compared with DP-EES [16,17]. Recent 3-year report of COMPARE II and NEXT showed the similar safety and efficacy outcomes between BP-BES and DP-EES, and partially dispelled concern regarding the safety of BP-BES [10,11]. However, there was limited data comparing safety and efficacy results of the BioMatrix BP-BES and Xience DP-EES in real world practice. An all-comer registry is needed to confirm safety and efficacy of BP-BES.

Concerning efficacy, the cumulative rate of TLF defined as a composite of cardiac death, target vessel-related myocardial infarction, or target lesion revascularization was similar between groups in our propensity score-matched population. This result was coincident with those of randomized trials compared with BP-BES and DP-EES, including COMPARE II and NEXT [10,11]. BP-BES could not show the improvement of efficacy outcome compared with DP-EES. With regard to safety, the benefit of BP-BES is expected beyond 1 year. The LEADERS trial showed lower risk of very late stent thrombosis in the BP-BES group compared to the durable polymer-coated sirolimus-eluting stent group [9]. In our study, definite or probable stent thrombosis occurred in 4 patients with BP-BES (0.4%) and in 4 patients with DP-EES (0.4%). Especially, very late stent thrombosis occurred in 1 patient with BP-BES (0.1%) and in 1 patient with DP-EES (0.1%). Very low rate of stent thrombosis were consistent with those of previous randomized trials [10,11]. In COMPARE II trial, stent thrombosis at 3 years occurred in only 9 patients with BP-BES (1.3%) and only 13 patients with DP-EES (1.4%) [11]. The 5-year rate of definite or probable stent thrombosis in DP-EES was also low (0.9%) in SORT OUT IV (Randomized Clinical Comparison of the Xience V and the Cypher Coronary Stents in Non-selected Patients With Coronary Heart Disease) trial [18]. There are several reasons why BP-BES failed to show superior safety compared to DP-EES. First, several randomized studies and registry showed that DP-EES still had an excellent safety [18–20], and comparison with BP-BES versus DP-EES might provide uncertain results. Second, median follow-up duration of 2 years may have been insufficient to assess long-term safety after BP-BES implantation compared with DP-EES implantation. Third, BP-BES has relatively thick strut (120 μm versus 81 μm) and polymer coating (10 μm versus 7.8 μm) compared with DP-EES. The possible benefit of biodegradable polymer might be offset by its thicker strut which is associated with higher risk of adverse clinical outcome. The optimal combination of ideal stent geometry, strut thickness, polymer coating technology, and drug in DP-EES plays a more important role in early-phase stent thrombogenecity than biodegradable polymer [17].

The present study could not avoid certain limitations associated with its observational nature. The use of BP-BES and DP-EES was at the discretion of the physician. Several confounding factors may have affected the results of our study. For example, DP-EES was used more frequently in the complex situation, possibly due to better deliverability of DP-EES [21]. Although we sought to reduce potential confounding using propensity score-matched analysis, we were not able to correct for the unmeasured variables. Second, clinical outcomes of BP-BES or DP-EES in present study were relatively low, and the power of the present study was low to draw any definite conclusion, especially stent thrombosis. However, we need to consider that this study was dedicated to comparing clinical outcomes of BioMatrix BP-BES and Xience DP-EES in real world setting. Randomized trials with long-term follow-up are needed to confirm equal safety and performance of both stents.

## Conclusion

The target lesion failure and stent thrombosis of BP-BES were similar to that of DP-EES in this propensity score-matched analysis of an observational registry.

## Supporting information

S1 Dataset Baseline characteristics of propensity score-matched population. (CSV)
